# Practicing traditional cultural games skills according to random and game-based practice schedules can improve gross motor skills performance

**DOI:** 10.3389/fpsyg.2024.1405635

**Published:** 2024-07-16

**Authors:** Jadeera Phaik Geok Cheong, Bahar Hussain

**Affiliations:** ^1^Faculty of Sports and Exercise Science, Universiti Malaya, Kuala Lumpur, Malaysia; ^2^Government Degree College Shewa (Swabi), Khyber Pakhtunkhwa, Pakistan

**Keywords:** contextual interference, game-based, gross motor skills, TGMD-2, traditional cultural games

## Abstract

The aim of this research was to examine the effects of using a random and game-based practice schedule for Traditional Cultural Games (TCG) skills on the performance of gross motor skills. Specifically, skills of two types of TCG, *Chindro*, an individual *TCG*, and *Pittu-Garam*, a team TCG, were practiced. 102 primary school children, ages seven to ten, attended a total of eighteen sessions of skill practice in 6 weeks, practicing TCG motor skills found in the game of Chindro or Pittu-Garam. For each TCG, participants were assigned to either random or game-based conditions, contributing to four experimental groups (Chindro random, Chindro game-based, Pittu random, and Pittu game-based). Gross motor skills performance was measured, before and after the experiment, according to the Test of Gross Motor Development-2. Particularly, the performance of six gross motor skills, Catch, Overhead Throw, Underhand Roll, Hop, Leap, and Jump, were assessed. Additionally, through a transfer test, the same skills were evaluated from a real-world game situation. In the skills performance test, all four groups significantly improved gross motor skills performance by the end of the experiment. However, there was no difference found between the groups based on game-based and random selection in the skills performance test and the game transfer test for both TCG. Practicing TCG skills according to high interference practice schedules, whether individually or in combination, improved gross motor skills performance. The study indicated that a game-based practice schedule could be a substitute for a random practice schedule when planning a training session involving high-interference practice schedules.

## 1 Introduction

In the motor learning literature, one of the recommendations for acquiring motor skills is that the learner practices according to practice schedules structured within the continuum of contextual interference (CI). At one end of the continuum, skills are practiced in a random order, classified as high CI, whereby it is necessary for the learner to change “randomly” between skills during practice. On the other end of the continuum, skills are practiced in a block order, classified as low CI, whereby the learner is required to practice one skill repeatedly before switching to another skill, and so on and so forth. Between these two ends of the continuum, skills can be practiced according to a moderate CI schedule and comprise practice conditions with different combinations of block and random practice trials.

Research into these different practice schedules has revealed a CI effect whereby research found that practice schedules with high interference were beneficial for learning, carried out in a variety of settings. In the laboratory, the CI effect was robust and many of the studies support a random practice schedule (Shea and Morgan, 1979; Shea and Zimny, 1983; Wu et al., 2011; Wright et al., 2016; Kim et al., 2018). Furthermore, the random practice schedule was also supported in the field setting (Goode and Magill, 1986; Aiken and Genter, 2018; Sharp et al., 2020). In the event where the CI effect was partially supported or not supported, moderate interference appeared to show positive benefits for acquisition and learning. For example, a gradually increasing practice schedule (Porter and Magill, 2010; Karimiyani et al., 2013; Yanci et al., 2013; Porter and Beckerman, 2016) was found to promote skill learning. A recent meta-analysis reported that a serial practice schedule was found to enhance skill acquisition (Lage et al., 2021). Regarding the type of skills that have been studied, the CI effect has been investigated in both non-sports (Jo et al., 2020; Jeon et al., 2021) and sports contexts (Goode and Magill, 1986; Porter and Magill, 2010; Cheong, 2011; Karimiyani et al., 2013; Yanci et al., 2013; Cheong et al., 2016; Aiken and Genter, 2018; Sharp et al., 2020; Menayo et al., 2022). In both these settings, most skills were practiced in isolation, in a closed skill environment. Only one study (Cheong et al., 2016) investigated the practicing of skills in a functional and open skill environment, using a game-based practice schedule that represented a form of random practice in a real-world settings. There was appear some support in favor of the CI effect when the sports skills were practiced in a real-world context (Cheong et al., 2016). Theoretically, the action plan reconstruction hypothesis (Lee and Magill, 1983; Lee et al., 1985) has been used to explain why moderate and high CI benefitted skill acquisition and learning, whereby the changes from practicing one skill to another forced the learner to forget details connected to the previous trial. Subsequently, the learner had to design a new plan of action that would help to strengthen memory and skill acquisition.

While the CI effect has been previously studied in lab and field studies involving a variety of motor skills, there is limited research involving skills from Traditional Cultural Games (TCG). Globally, TCG has been studied in various fields, such as sociology (Kovačević and Opić, 2014; Lavega et al., 2014; Anastasovski et al., 2016), psychology (Trajkovik et al., 2018), and even in sports and exercise (Akbari et al., 2009; Abdullah et al., 2013). In the context of sport and exercise science in particular, participation in TCG were reported to be beneficial for physiological health and fitness (Akbari et al., 2009; Abdullah et al., 2013). Motor skills are an important component of children’s fitness (Barnett et al., 2018) and support the development of more complex skills (Basman, 2019). It was previously reported that motor skills need to be actively taught and should not be assumed that children would pick up the skills naturally as they grow and develop across time (Hands, 2012; Mukherjee et al., 2017).

Previously, solely one study had examined the CI effect using TCG skills (Hussain and Cheong, 2022). The participants of that study had practiced team TCG skills according to different practice schedules ranging from low to moderate and high interference. The study found that the high CI groups (random) outperformed the low CI (block schedule) and moderate CI (gradually increasing schedule) when assessed after 18 sessions of practice, supporting the CI effect involving team TCG skills, and justifying the use of high CI over low or moderate CI for this study. However, it is not known if this effect can be generalized from all types of TCG skills, including skills from TCG that are played individually. In individual TCG, victory or failure depends completely on the individual, making the situation more challenging for an individual TCG compared to a team TCG. As such, there could be an additional element of interference arising from practicing individual TCG skills, other than the inter-task and intra-task interference, both of which have been reported to have a great influence on the CI effect (Goode and Magill, 1986; Aiken and Genter, 2018; Sharp et al., 2020). It is not known if this additional interference may influence high CI schedules. Therefore, the aim of this research was to examine the effects of practicing individual and team TCG skills according to high CI practice schedules on gross motor skills performance. Specifically, the effectiveness of random and game-based practice schedules was compared in learners practicing individual (*Chindro*) and team (*Pittu-Garam*) TCG skills. The random practice represented high interference in an isolated, closed skill environment while the game-based training protocol represented high interference in a functional, real-world environment. It was hypothesized that practicing TCG skills, according to high interference schedules in both environments, would improve gross motor skill performance.

## 2 Materials and methods

### 2.1 Participants

According to the G-Power sample size calculator (a-priory power analysis for *f*-test family), and based on a meta-analytic Study by Brady (2004) which reported an effect size of 0.38, using power = 0.80, and α err value = 0.05, 80 primary school children (20/Group) in total were required. To account for dropouts, the researcher increased the number of participants in each group from five to six. Therefore, in total, 102 primary school children between the ages of 7 to 10 were selected through convenience sampling technique from two schools situated in the rural area of Khyber Pakhtunkhwa province of Pakistan. The children’s mean age was 8.17 ± 1.34 years. Participants with prior TCG experience in the chosen TCG of this study were excluded. The parents of the children had signed a copy of the informed consent form according to the ethical guidelines of the University of Malaya Research Ethics Committee (Reference number: UM. TNC2/UMREC_1047).

### 2.2 Practice tasks and experimental groups

Two TCGs were involved in the experiment. The first TCG, *Chindro* (a type of hopscotch), is an individual TCG that involves Throwing, Hopping, Leaping, and Jumping skills. The second TCG, *Pittu-Garam* (seven stones) is a team TCG that involves Catching, Overhead Throw, Underhand Throw, and Leaping skills. Participants of both the TCGs were assigned randomly to four experimental groups, each with high levels of CI in functional or nonfunctional environments. Participants were assigned randomly to four experimental groups, each with distinct levels of CI: (a) *Chindro* random (High CI in the nonfunctional environment), (b) *Chindro* game-based (High CI in the real-world setting with the functional environment), (c) *Pittu* random (High CI in the nonfunctional environment), and (d) *Pittu* game-based (High CI in the real world setting with the functional environment).

#### 2.2.1 Tasks for *Chindro* group

**Figure 1:**
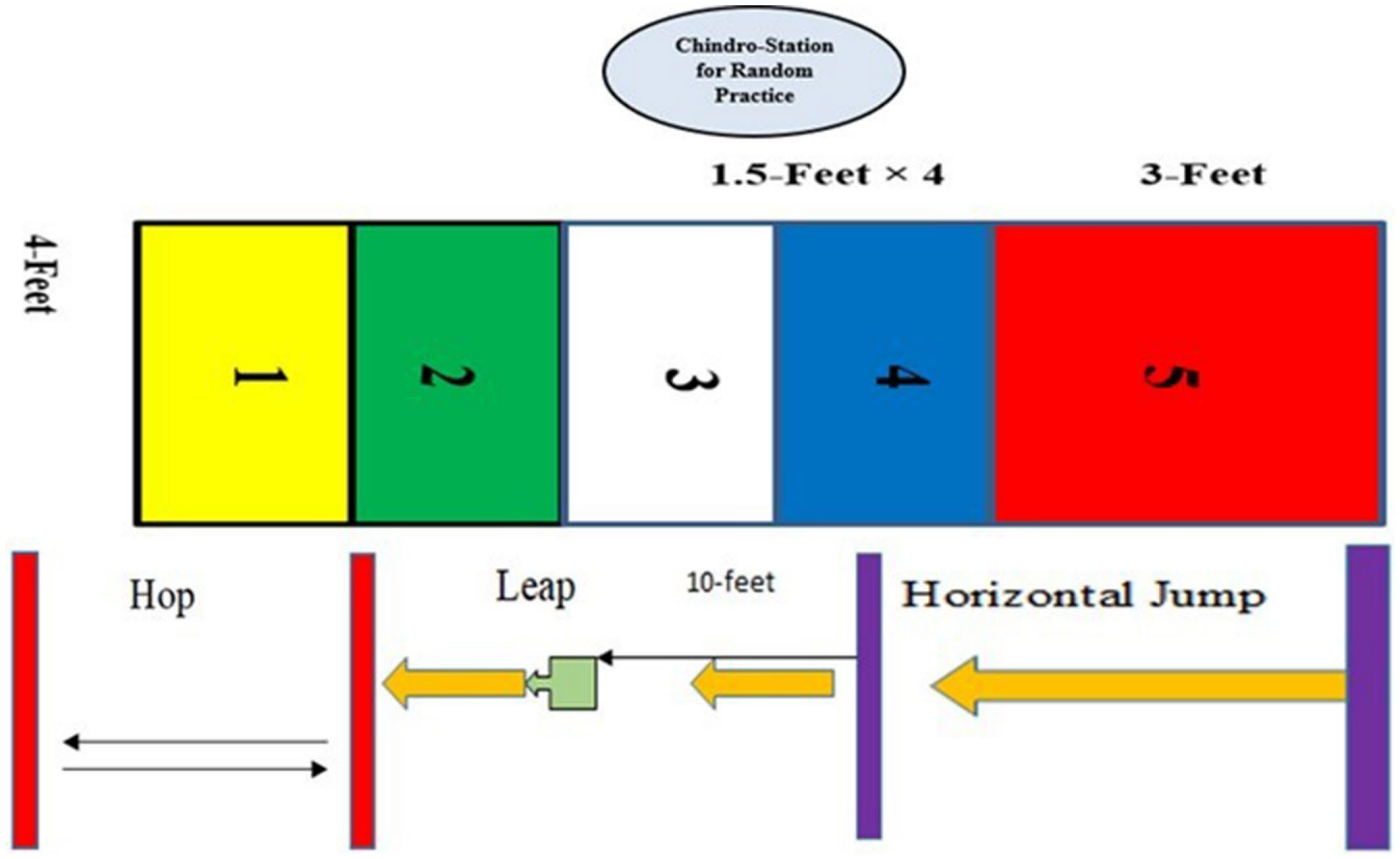
Layout of random practice for Chindro.

Participants in *Chindro* were divided into two subgroups: *Chindro* random and *Chindro* game-based. *Chindro* Random group practiced four motor skills, i.e., Throwing tekre (Underhand Throw of a flat round stone), Hop, Leap, and Jump. *Chindro* randomly practiced all four skills in isolation, in random sequence, with no more than two consecutive trials of the same skill. The layout for *Chindro* random is shown in Figure 1. The experimental program for *Chindro* random is shown in Table 1.

**Table 1:** Order of practice for both the Games (Chindro and Pittu-Garam) in each session.

Order of practice for Chindro random and Chindro game-based on each session		
Practice group	Chindro random	Chindro game-based
Activity	Throwing, Hop, Leap, Jump	Individual play
Warm-up	4 min	4 min
Order of practice	T, H, L, J, J, T, H, L, L, J……. 16 trials for each skill in random order, avoiding more than two consecutive trials of the same skill.	Playing Chindro with individual play
Cool-down	4 min	4 min
Order of practice for Pittu random and Pittu game-based on each session		
Practice group	Pittu random	Pittu game-based
Activity	Catch, Overhead throw, underhand throw, Leap	Team play
Warm-up	4 min	4 min
Order of practice	C, O, U, L, L, C, O, U, U, L……. 16 trials for each skill in random order, avoiding more than two consecutive trials of the same skill.	Playing pittu with four (4) players/group
Cool-down	4 min	4 min

**Figure 2:**
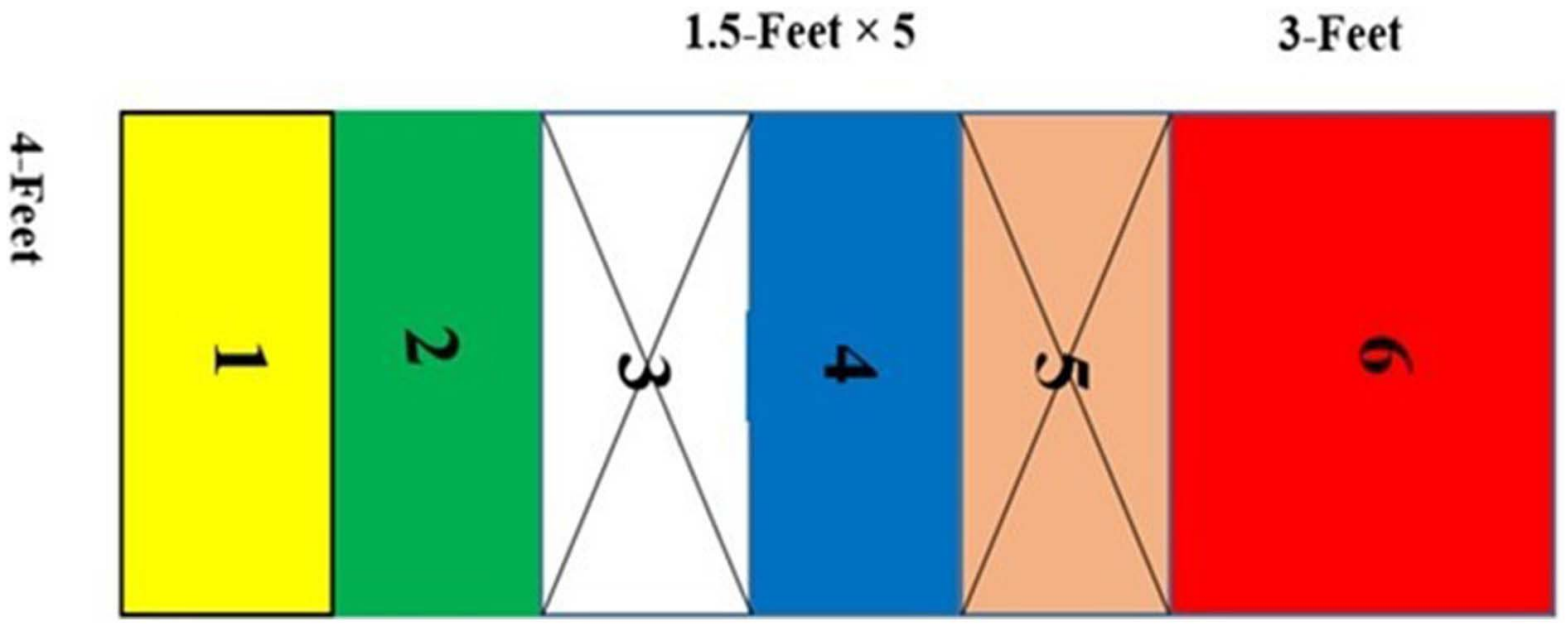
Layout of game-based training for Chindro.

Alternately, participants of the *Chindro* game-based practiced all four skills in the course of playing a total of four games of *Chindro*. The practice space for the *Chindro* game-based training group was 10 and a half feet long and 4 feet broad. This rectangular space was divided into six squares, each about a foot and a half wide, with the last one measuring 3 feet. For training purposes, the third and fifth squares were manipulated and closed. The first is known as Yakka, the second is known as Dwaka, the third (close) is known as Dreka, the fourth is known as Salorka, and the fifth (close) is known as Sengla, all one and a half feet wide and the sixth and final one is known as Damo, which is 3 feet wide (see Figure 2 for layout). The experimental program for the *Chindro* game-based is shown in Table 1.

#### 2.2.2 Tasks for *Pittu Garam* group

Participants of *Pittu-Garam* were divided into two subgroups: *Pittu* random and *Pittu* game-based.

**Figure 3:**
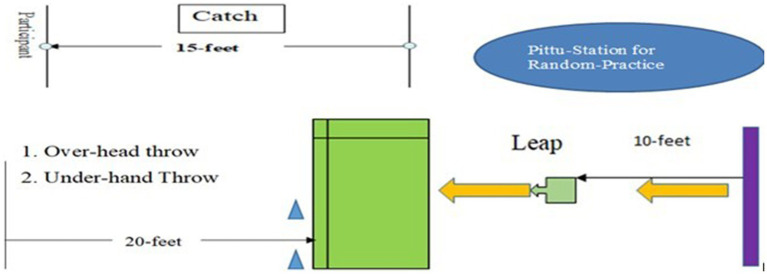
Layout of random practice for Pittu-Garam.

*Pittu* Random group practiced four GMS, i.e., Catch, Overhead Throw, Underhand Throw, and Leaping. Participants practiced all four skills independently, in random order, with no more than two consecutive trials of the same skill. The layout for the random training of the *Pittu* random group is shown in Figure 3. The experimental program for *Chindro* random is shown in Table 1.

**Figure 4:**
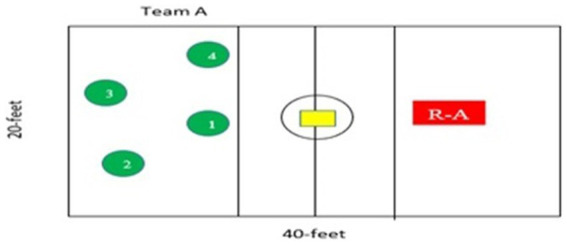
Layout of game-based training for Pittu-Garam.

Alternately, participants of *Pittu* game-based practiced all four skills in the course of playing a total of four rotations of the *Pittu* game-based. The Pittu game-based training group’s rectangle was modified to be 20 feet wide and 40 feet long. This rectangle was split into two equal pieces measuring 20 feet by 20 feet. Two parallel lines were drawn 6 feet from the centerline along both sides. The ball was to be thrown on one side by the research assistant and on the other by four participants stationed clockwise (see Figure 4 for layout). Participants practiced all four skills while playing a game of *Pittu-Garam*, in the experimental program for *Pittu* game-based as shown in Table 1.

### 2.3 Measures and instrumentation

#### 2.3.1 Motor skill performance test

The Test of Gross Motor Development (TGMD-2; Ulrich, 2000) was used for assessing six gross motor skills: Catch, Overhead Throw, Underhand Roll, Hop, Leap, and Horizontal Jump. Two trials of each skill were performed and the scores of both trials were combined to form a total score. According to the TGMD-2 manual, the procedural score for Catch is 6, Overhead Throw is 8, Underhand Roll is 8, Hop is 10, Leap is 6, and Horizontal Jump is 8. Addressing criterion validity, the TGMD-2 showed a moderate to strong correlation with the basic generalization subset of comprehensive scales of students’ skills (*r* = 0.63 for locomotor; *r* = 0.41 for object control; and *r* = 0.63 for the composite; Ulrich, 2000). Furthermore, it was reported (Ulrich, 2000) that the model proposed is validated by the data according to the findings of confirmatory factor analysis (CFA). The goodness of fit indexes was good relative to chi-square 5.29 and the goodness of fit index (GFI), adjusted goodness of fit index (AGFI), and Trucker-Lewis Index (TLI) ranged from 0.90 to 0.96.

A sample of 30 participants was selected and they performed two tests delayed by 1 week. The reliability of the same rater over two trials using the intra-class correlation coefficients for the Hop 0.93 (95% CI = 0.86, 0.96), Leap 0.90 (95% CI = 0.79, 0.95), Jump 0.95 (95% CI = 0.90, 0.97), Catch 0.95 (95% CI = 0.90, 0.97), OHT 0.93 (95% CI = 0.86, 0.97), and UHR was 0.96 (95% CI = 0.91, 0.98) respectively, showing good reliability overall. Moreover, for the sum of four skills (Hop, Leap, Jump, and Underhand Roll) of the Chindro game the intra-class correlation coefficient was 0.94 (95% CI = 0.88, 0.97), and for the sum of four skills (catch, overhead throw, underhand roll, and leap) of Pittu game the intra-class correlation coefficients were 0.96 (95% CI = 0.92, 0.98). Furthermore, the intra-class correlation coefficients for the sum of two skills (Leap and UHR) was 0.94 (95% CI = 0.87, 0.97).

For *Chindro* groups, four skills (Hop, Leap, Jump, and Underhand Roll) were measured, and the scores for all four skills were combined to form a composite score between 0 and 32. Whereas for *Pittu* groups, four skills (catch, overhead throw, underhand roll, and leap) were measured, and the scores for four skills were combined to form a composite score between 0 and 28. Additionally, for both *Chindro* and *Pittu* groups (combined), two skills (Leap and Underhand Roll) were also measured, as these two skills were common in both TCG. The minimum composite score was 0 and the maximum was 14.

#### 2.3.2 Game transfer test

Both the *Chindro* and *Pittu-Garam* groups performed separate Game Transfer Tests.

For *Chindro,* all participants played against each other. Each two-player played one match (1*1) against each other, within and between *Chindro* random and *Chindro* game-based groups according to the local rules and regulations. For every individual, the lowest possible score was 0, and the highest possible score was 10.

As for *Pittu-Garam*, there were 24 participants in each group. Six teams of four players were made up from each group. There were four players in each team, and each team played one match against another team. 4 × 4 matches were played between and within the *Pittu* random and *Pittu* game-based groups for all the teams according to local rules and regulations, on a standard ground of 40 × 80 feet. The winning team was the one to reach seven points first, and each match’s final score for each side was recorded. For each team, the lowest possible score was 0, and the highest possible score was 7.

### 2.4 Procedures

The study comprised 6 weeks and involved 18 practice sessions. A study introduction was presented 1 day before to the start of the practice sessions, which included a presentation of the four tasks (Underhand Throw, Hop, Leap, and Jump) to the *Chindro* groups and four tasks (Catch, Overhead Throw, Underhand Throw, and Leap) to *Pittu-Garam* groups. The participants also received written information for each skill. The participants were also shown a video highlighting both the games and skills of Pittu-Garam and Chindro. The initial session was ended with a Motor Skill Performance Pre-test. There were two trials of each skill (Underhand Throw, Hop, Leap, and Jump) in one session of the *Chindro* group in the Pre-test. Similarly, there were two trials of each skill (Catch, Overhead Throw, Underhand Throw, and Leap) of the *Pittu-Garam* group in the Pre-test. Each skill was recorded on camera and evaluated later by the principal investigator.

The participants attended 18 practice sessions according to the tasks specified to their respective group. At the start of each session, all four groups were given verbal instructions and observational demonstrations of each skill and game. After completing the last practice session, the Motor Skill Performance Post-test was carried out ensuring the Pre-test’s format. Game Transfer Test was administered the day after the completion of the Motor Skill Performance Post-test.

### 2.5 Statistical analyses

The Statistical Package for Social Sciences (SPSS) version 23 was used to analyze the data. For both TCG, motor skill performance scores were analyzed using 4 groups (*Chindro* random, *Chindro* game-based, *Pittu* random, *Pittu* game-based) × 2 time periods (Pre-test, Post-test) split-plot analysis of variance (SPANOVA) for Leap and Underhand Roll.

For the *Chindro* group, the overall performance score was analyzed using 2 groups (*Chindro* random, *Chindro* game-based) × 2 time periods (Pre-test, Post-test) split-plot analysis of variance (SPANOVA) for Hop, Leap, Jump, and Underhand Roll. Additionally, for the *Pittu-Garam* group, the overall performance score was examined using 2 groups (*Pittu* random, *Pittu* game-based) × 2 time periods (Pre-test, Post-test) split-plot analysis of variance (SPANOVA) for Catch, Overhead Throw, Underhand Roll, and Leap.

For the Transfer Test, an independent sample *t*-test was conducted to compare the scores of both *Chindro* groups (*Chindro* random, *Chindro* game-based) and both *Pittu* groups (*Pittu* random, *Pittu* game-based), respectively. In all cases, the Bonferroni adjustment had been utilized for the *post hoc* comparisons, and the cutoff point of significance was set at alpha = 0.05 for each analysis.

## 3 Results

### 3.1 Demographic information

A total of 61 male and 41 female students (Mean age 8.17 ± 1.34 years) participated in the study. The breakdown of the participants according to their groups, age, and gender is shown in Table 2.

**Table 2:** Distribution of participants by gender and practice group (*N* = 102).

Participant groups	Mean age	SD	Gender		Total
			Male	Female	
Chindro random	8.27 years	1.09	15	10	25
Chindro game-based	8.31 years	1.20	16	10	26
Pittu random	7.72 years	1.79	13	11	24
Pittu game-based	8.32 years	1.17	17	10	27
Total	8.17 years	1.34	61	41	102

### 3.2 Motor skill performance test scores

For all four groups (*Chindro* random, *Chindro* game-based, *Pittu* random, *Pittu* game-based), the sum of the scores for the two skills, Leap and Underhand Roll, were calculated. There was a statistically significant main effect for time, *F*_(1, 98)_ = 186.662, *p* < 0.01, $\eta_{\mathrm{Partial}}^{2}$ = 0.656, indicating that overall, all four groups improved at the end of the practice sessions, compared to the Pre-test. Meanwhile, the main effect for group, [*F*_(3, 98)_ = 0.158, *p* = 0.924, $\eta_{\mathrm{Partial}}^{2}$ = 0.005], and the time and group interaction [*F*_(3, 98)_ = 2.541, *p* = 0.061, $\eta_{\mathrm{Partial}}^{2}$ = 0.072] were not significant.

For the *Chindro* Groups (*Chindro* random, *Chindro* game-based), the sum of the scores for four skills, Hop, Leap, Jump, and Underhand Roll, were calculated. There was a statistically significant main effect for time [*F*_(1, 48)_ = 174.585, *p* < 0.01, $\eta_{\mathrm{Partial}}^{2}$ = 0.784], indicating that both groups improved scores at the end of the practice sessions, as compared to the Pre-test. Meanwhile, the main effect for the group [*F*_(1, 48)_ = 0.055, *p* = 0.815, $\eta_{\mathrm{Partial}}^{2}$ = 0.001], and the time and group interaction, [*F*_(1, 48)_ = 0.144, *p* = 0.706, $\eta_{\mathrm{Partial}}^{2}$ = 0.003] were not significant.

For *Pittu-Garam* Groups (*Pittu* random, *Pittu* game-based) the sum of the scores for the four skills, Catch, Overhead Throw, Underhand Roll, and Leap, were calculated. There was a statistically significant main effect for time [*F*_(1, 49)_ = 302.604, *p* < 0.01, $\eta_{\mathrm{Partial}}^{2}$ = 0.861], indicating that both groups improved scores at the end of the practice sessions, as compared to the Pre-test. Meanwhile, the main effect for the group [*F*_(1, 48)_ = 0.185, *p* = 0.669, $\eta_{\mathrm{Partial}}^{2}$ = 0.004], and the time and group interaction [*F*_(1, 48)_ = 0.374, *p* = 0.544, $\eta_{\mathrm{Partial}}^{2}$ = 0.008] were not significant. The mean and standard deviation of Skill Performance Test scores for each group during the Pre-test and Post-test are shown in Table 3.

**Table 3:** Means and standard deviations of skill performance scores for each group at Pre-test and Post-test.

Skill/Group	N	Pre-Test		Post-Test	
		*M*	*SD*	*M*	*SD*
Means and standard deviations of skill performance scores (Leap and Underhand Roll) for each group (Chindro random, Chindro game-based, Pittu random, Pittu game-based) at Pre-test and Post-test. (14)					
Chindro random	25	6.56	(2.63)	09.28	(1.95)
Chindro game-based	26	6.54	(2.67)	09.73	(1.91)
Pittu random	24	5.50	(2.75)	10.04	(1.49)
Pittu game-based	27	6.48	(2.76)	09.51	(1.65)
Means and standard deviations of skill performance scores (Hop, Leap, Jump, and Underhand Roll) for Chindro random and Chindro game-based groups at Pre and Post-test. (32)					
Chindro random	25	14.96	(4.44)	22.12	(3.19)
Chindro game-based	26	14.92	(4.80)	21.68	(3.61)
Means and standard deviations of skill performance scores (Catch, Overhead Throw, Underhand Roll, and Leap) for Pittu random and Pittu game-based groups at Pre and Post-test. (28)					
Pittu random	24	11.50	(3.84)	21.70	(2.11)
Pittu game-based	27	12.15	(3.93)	21.74	(2.51)

### 3.3 Game transfer test scores

For the Game Transfer Test, non-significant improvements in GMS were observed during *Chindro* skills (*p* = 0.63, 95% CI: −0.58, 0.96) and *Pittu-Garam* skills (*p* = 0.20, 95% CI: −4.35, 1.02). Means and standard deviations of random and game-based scores for each group of the two TCGs, *Chindro* random and *Chindro* game-based as well as *Pittu* random and Pittu game-based are shown in Table 4.

**Table 4:** Means and standard deviations of Game-based scores for each group of the two TCGs: (Chindro random, Chindro game-based) and (Pittu random, Pittu game-based).

	Group	*N*	Mean	Std. Deviation
Score	Chindro randomChindro game-basedPittu random	27	5.85	1.54
		27	5.67	1.27
		6	4.17	2.32
	Pittu game-based	6	5.83	1.83

## 4 Discussion

In view of the importance of motor learning and development, this study intended to figure out the effect of practicing individual (*Chindro*) and team (*Pittu-Garam*) TCG skills according to two different practice schedules (random and game-based) on primary school children’s acquisition of GMS.

The results showed that, overall, participants from all four groups improved their performance in all motor skill performance tests. The motor skill performance test scores at the end of the 6-week practice session were significantly better compared to the Pre-test scores. Specifically, both the random and game-based groups in both TCGs had significantly higher scores upon completion of the experiment. This finding supports the CI literature that practice schedules with high interference were effective for learning motor skills (Lee and Magill, 1983; Wu et al., 2011; Wright et al., 2016; Kim et al., 2018). When practicing according to a random schedule, the learner practiced each skill in isolation and was required to switch between skills “randomly” throughout practice. Meanwhile, when practicing according to a game-based schedule, the learner was also required to switch between skills randomly but in functional environment. In both conditions, the switching between skills caused a high inter-task interference. The theories of increased forgetting (Lee et al., 1985) or deep processing (Shea and Zimny, 1983) both may explain the benefits of practicing with high CI. The action plan reconstruction hypothesis (Lee et al., 1985) stated that high levels of CI required the learner create a plan of action for the forthcoming skill variation each time a skill was executed. Alternatively, according to the elaboration hypothesis (Shea and Zimny, 1983) learners with high CI were required to elaborate and make comparisons and contrasts between any memory of prior skills that could assist them perform the current skill. Both theories may explain why the participants of the random and game-based practice schedules improved their motor skills. However, the CI effect in skills variations is better explained by the action plan reconstruction hypothesis so, it is important to mention that the current study explains better by the action plan reconstruction hypothesis rather than the elaboration hypothesis.

Although improvements were found across time for all four groups in the motor skill performance test, no differences were found between random and game-based groups for both TCGs in the motor skill performance test and the game transfer test. The results of this study were consistent with two previous studies comparing random and game-based practice schedules. Both studies suggested that game-based practice was a possible alternative to a random practice schedule for learning field hockey skills (Cheong et al., 2016) and traditional games skills (Hussain and Cheong, 2022), respectively. Whilst both studies found that the high CI groups performed better than the low or moderate CI groups, no differences were found between both high CI groups. There are two possible reasons why there were no differences between random and game-based groups in the current study. Firstly, even though the random groups were practicing skills in isolation, they had knowledge that the skills were from TCG and could relate the skills to the game to give it meaning and enjoyment. Secondly, since both groups were practicing according to a high-interference practice schedule, the CI effect was present and benefited both groups due to the high interference between trials.

On the aspect of TCG, previously the use of TCGs was found to be beneficial in social, ethnic (Lavega et al., 2014; Anastasovski et al., 2016), and cultural (Bashir and Zain-Ul-Wahab, 2018), frameworks. Furthermore, a few studies in the field of exercise science have examined TCG and found it beneficial for physical health (Akbari et al., 2009; Abdullah et al., 2013). The results of this study were in line with the previous studies and illustrated the benefits of learning TCG skills for the development of motor skills of children, especially those aged between 7 to 10 years. Furthermore, the results contributed to literature involving TCG in the field of exercise science in general, and motor learning in particular. While the TCGs used in this study are native to Pakistan, there are similarities with other TCGs in terms of the motor skills involved. For example, Chindro is akin to Hopscotch and involves hopping, jumping and throwing. Hopscotch was a prominent game in England and had spread over the whole of Europe, appearing under numerous aliases in England, Scotland, Ireland, France, Spain, Italy, Sweden, Finland, Portugal and other subcontinents (Bashir and Zain-Ul-Wahab, 2018). Similarly, Pittu-Garam helps to develop and hone hopping, leaping, throwing, strategy building skills, and teamwork in children (Sahay, 2013; Bashir and Zain-Ul-Wahab, 2018) with these motor skills found in other TCGs or physical activities. It was reported that in different regions of the world the differences between games concern only names or some rules, such as in many games with stones and sticks (Piech and Cieślinski, 2007).

Finally, this study has some limitations. In the current study, an experiment to enhance GMS was conducted using two TCGs (*Chindro* and *Pittu-Garam*). While both games are popular in the Pakistani province of Khyber Pakhtunkhwa, these games may not be applied to all children and not all children partaking in TCGs from various provinces, cultures, and nations may experience the same improvements in GMS. This is due to the various TCGs available worldwide, each with a unique set of rules, apparatus, and playing surfaces depending on the environment and location. It has been reported that geographical area, the environment, and ecological aspects of how a child grows up and living have a significant impact on the rate and level of their motor development (de Barros et al., 2003). Additionally, we also recruited our participants through convenience sampling, and it is possible that our sample does not fully represent the population.

In summary, practicing TCG skills according to high CI schedules, such as random and game-based schedules, both are effective for facilitating the learning and development of GMS of students aged 7 to 10 years. This finding supports the CI literature that practice schedules with high interference were effective for learning motor skills (Lee and Magill, 1983; Wu et al., 2011; Wright et al., 2016; Kim et al., 2018). Additionally, the theory of Schmidt (1975) indicated that children, who are at the beginning of schema formation, should see more effects than adults (Merbah and Meulemans, 2011). Children acquire and grow in a wide range of GMS in the early years through both planned and unplanned experiences in secure and supportive environments that are developmentally appropriate (Brian et al., 2020).

## 5 Conclusion

Practicing individual or team TCG skills according to high CI schedules such as random and game-based practice was beneficial for learning and developing GMS in primary school children, specifically children between 7 and 10 years of age. Whether the randomization of skills was practiced in a nonfunctional environment or a functional environment, no differences were found in motor skill performance. The CI effect was supported, and game-based practice could be suitable as an alternative practice schedule for learning and transferring gross motor skills. Moving forward, we recommend that long-term effects are investigated to strengthen the outcomes of this study.

## Data availability statement

The raw data supporting the conclusions of this article will be made available by the authors, without undue reservation.

## Ethics statement

The studies involving humans were approved by University of Malaya Research Ethics Committee (Reference number: UM. TNC2/UMREC_1047). The studies were conducted in accordance with the local legislation and institutional requirements. Written informed consent for participation in this study was provided by the participants’ legal guardians/next of kin.

## Author contributions

JC: Conceptualization, Methodology, Project administration, Software, Supervision, Validation, Visualization, Writing – review & editing. BH: Conceptualization, Data curation, Formal Analysis, Methodology, Software, Writing – original draft.
